# CODEX: a next-generation sequencing experiment database for the haematopoietic and embryonic stem cell communities

**DOI:** 10.1093/nar/gku895

**Published:** 2014-09-30

**Authors:** Manuel Sánchez-Castillo, David Ruau, Adam C. Wilkinson, Felicia S.L. Ng, Rebecca Hannah, Evangelia Diamanti, Patrick Lombard, Nicola K. Wilson, Berthold Gottgens

**Affiliations:** 1 Department of Haematology, Wellcome Trust-MRC Cambridge Stem Cell Institute & Cambridge Institute for Medical Research, Cambridge University, Cambridge CB2 0XY, UK; 2 Wellcome Trust-MRC Cambridge Stem Cell Institute, University of Cambridge, Cambridge, CB2 1QR, UK

## Abstract

CODEX (http://codex.stemcells.cam.ac.uk/) is a user-friendly database for the direct access and interrogation of publicly available next-generation sequencing (NGS) data, specifically aimed at experimental biologists. In an era of multi-centre genomic dataset generation, CODEX provides a single database where these samples are collected, uniformly processed and vetted. The main drive of CODEX is to provide the wider scientific community with instant access to high-quality NGS data, which, irrespective of the publishing laboratory, is directly comparable. CODEX allows users to immediately visualize or download processed datasets, or compare user-generated data against the database's cumulative knowledge-base. CODEX contains four types of NGS experiments: transcription factor chromatin immunoprecipitation coupled to high-throughput sequencing (ChIP-Seq), histone modification ChIP-Seq, DNase-Seq and RNA-Seq. These are largely encompassed within two specialized repositories, HAEMCODE and ESCODE, which are focused on haematopoiesis and embryonic stem cell samples, respectively. To date, CODEX contains over 1000 samples, including 221 unique TFs and 93 unique cell types. CODEX therefore provides one of the most complete resources of publicly available NGS data for the direct interrogation of transcriptional programmes that regulate cellular identity and fate in the context of mammalian development, homeostasis and disease.

## INTRODUCTION

One of the fundamental questions in biology is how a single fertilized egg cell faithfully develops into a multicellular organism containing specialized organs capable of homeostasis and regeneration, while the genomic content within each cell remains essentially unchanged. Cell-type specific transcriptional and chromatin landscapes are critical determinants of the global gene expression patterns that define cell identities and fate choices (1). As key regulators of these processes, transcription factors (TFs) are thought to act combinatorially to confer context-specific activities responsible for orchestrating global gene expression patterns that drive stem cell self-renewal, proliferation, homeostasis, cell differentiation and specification (2). A unified understanding of these complex processes is still in its infancy. Two of the most studied systems of mammalian development are the haematopoietic system and embryonic stem (ES) cells (3–5). The haematopoietic system is also of particular interest in the context of disease, where transcriptional dysregulation is known to drive numerous haematological malignancies (6,7).

Recent advances in next-generation sequencing (NGS) have allowed genome-wide analysis of TF binding and histone modifications (by chromatin immunoprecipitation coupled to high-throughput sequencing; ChIP-Seq), identification of open regions of chromatin (by DNase-Seq) and transcriptomic analysis (by RNA-Seq) (8). Such technologies have the potential to drive key advances in our understanding of mammalian development, homeostasis and disease. Both large international consortia (such as ENCODE and BLUEPRINT) (9,10) and numerous individual laboratories are effectively generating and releasing such genome-wide datasets into the public domain. Current repositories for raw NGS data include the Gene Expression Omnibus (GEO) (11), ArrayExpress (12) and the DNA Data Bank of Japan (DDBJ) (13). These datasets provide a wealth of information, for both large-scale whole-genome meta-analyses and the study of single genomic loci.

However, the multi-centre nature of this huge data generation effort has had several unintended side effects: (i) the bioinformatic processing and analysis necessary to provide informative and biologically relevant insights from such experiments are not uniformly standardized or integrated, (ii) no public repository provides instant NGS data visualization, (iii) the large size of such NGS datasets (raw RNA-Seq datasets can be 100 GB) is prohibitive for the in-house processing necessary for visualization and/or further analysis without dedicated computer hardware or bioinformatics expertise and finally (iv) annotation of publicly available NGS data is often incomplete or non-intuitive, limiting simple data interpretation. These current failures significantly reduce the utility of such data to the wider research community.

In an effort to bridge this gap between the vast amounts of publicly available NGS raw data and end-user friendly information, we have developed CODEX (http://codex.stemcells.cam.ac.uk/), a database of NGS experiments including ChIP-Seq, RNA-Seq and DNase-Seq. CODEX provides uniformly processed data as well as online resources for NGS data visualization and bioinformatics analysis. Most importantly, CODEX uses a standardized bioinformatics-processing pipeline for all NGS datasets, and the details of each sample are manually curated to provide key information. CODEX currently includes over 1000 uniformly processed NGS datasets that can be easily viewed, interrogated and compared by the general scientific community, for both quick and informative comparisons as well as large-scale meta-analyses.

The current focus of CODEX is to unify NGS data for the haematopoietic system and ES cells. CODEX therefore encompasses two specialized compendia: one focused on blood cells (HAEMCODE), and a second focused on data from ES cells (ESCODE). In addition, other relevant cell types are also held in CODEX. To date, CODEX comprises over 1000 samples selected for maximal genomic coverage, with 221 unique TFs and 93 unique cell types across both human and mouse samples. CODEX therefore currently provides one of the most complete resources to directly interrogate transcriptional regulation of mammalian development, homeostasis and disease.

## DATA SOURCES AND PROCESSING

**Figure 1.**
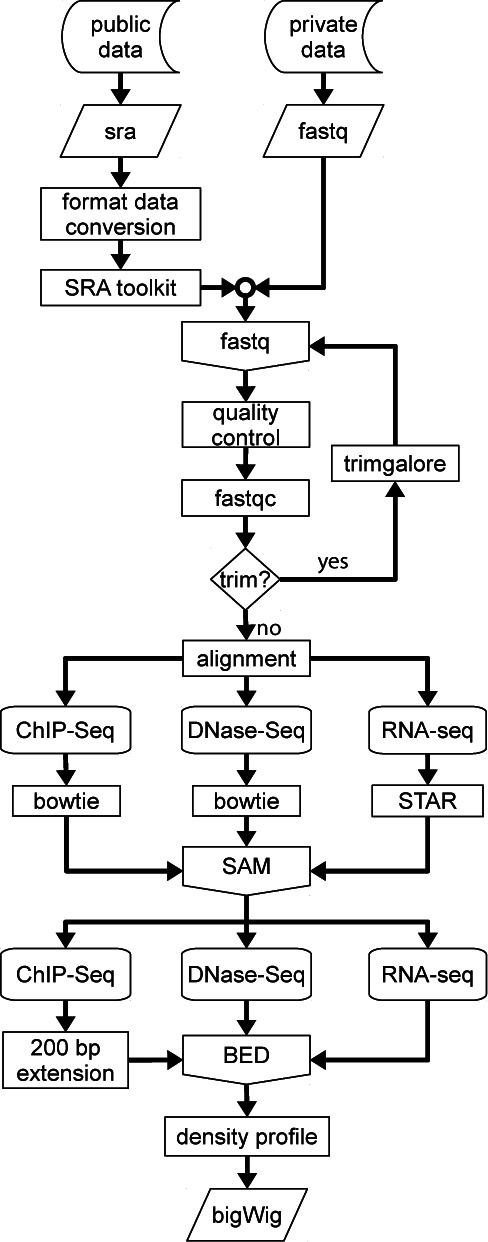
Flow diagram of the CODEX processing pipeline for ChIP-Seq, RNA-Seq and DNase-Seq. Data is downloaded from GEO and converted to fastq (in-house experiments are directly provided in this format). A quality test is performed and adapters and overrepresented sequences are removed from the raw reads. Trimmed sequences are then aligned and the resulting SAM file is converted to a BED format file from which a density profile is computed.

CODEX is built on bioinformatics pipelines that uniformly process all relevant publicly available NGS experiments (Figure 1), allowing NGS experiment integration and direct comparison. Additionally, sample details are manually curated to provide users with key information to understand each experiment.

### Data sources

Raw NGS data are stored on and can be downloaded from GEO and ArrayExpress in a standardized common format as Sequence Read Archive data files (SRA) (14). Although GEO and ArrayExpress also store aligned data files and processed data for some experiments, such processing is non-standardized and therefore cannot easily be used for downstream comparison. These databases have a common basic structure, with each experiment being recorded under a unique sample identifier and grouped together into a series record with the remainder of the experiments in the same study. The same hierarchical structure is used to record series and individual samples in CODEX, with each public experiment from GEO or ArrayExpress being recorded in our database using both the sample and the series accession.

### Web crawling of functional genomic repositories

To ensure CODEX remains up-to-date with newly published datasets we have developed a web crawler that searches the GEO database and reports any new samples of relevance to CODEX. This robot not only retrieves the GEO sample and series accessions, but also scans other databases (such as NCBI SRA browser, BioProject and BioSample) (15) to collect information and details of the experiments. The web crawler uses a text-mining approach to automatically classify each sample according to experiment type (TF ChIP-Seq, histone ChIP-Seq, DNase-Seq and RNA-Seq) and where applicable, assigns the sample to the corresponding specialized repository within CODEX. Each experiment is then annotated with the official name of the factor (where applicable) and the corresponding unique EntrezGene identifier. The shortlisted experiments are then manually reviewed, and any inappropriate candidates on the list are discarded. This method results in an average of 50 new samples per week.

In addition to this semi-supervised method, individuals can also report any experiment or study from GEO and ArrayExpress. All requested analyses are vetted and if suitable for CODEX are added to the processing queue.

### Manual curation of sample details

Although valuable experimental information is provided by users at the time of GEO submission, such details are often poorly curated or incomplete. Such available information is collected by CODEX via its automated text-mining step. This information alongside details from relevant publications is then used to manually curate each sample added to CODEX. This is a time consuming but critical step in the processing pipeline because it provides end users with the information for each experiment, allowing users to distinguish between cell types and culture conditions that can otherwise be difficult to determine.

CODEX also provides the following hierarchically structured information for each sample to allow users to easily search, choose, compare and contrast different samples. Each of these fields is individually manually curated:

- General cell type: broadly defines the sample.
- Cell subtype: distinguishes cell lines from primary cells and includes the specific cell type name. This field also contains treatment type so users are aware that specific treatments have been utilized.
- Tissue Ontology classifier from the BRENDA database (BTO); hierarchically organizes cell types and cell lines under a generic category corresponding to the rules and formats of the Gene Ontology Consortium (16).
- Additional details: includes (where appropriate) any special culture conditions or drug treatments, as well as human patient cell line chromosomal abnormalities.

### Standardized data processing

Central to CODEX is its processing algorithm, which removes one of the main difficulties users may find when comparing publicly available data. Raw NGS data mapped to different genome builds are not directly comparable and require a good knowledge of the plethora of bioinformatics tools to perform this task. Even when mapped to the same genome, the software used or processing details may differ, which prevents direct comparison.

To facilitate the ease of use of the processed data within CODEX, we have developed our own NGS pipeline based on open-source and widely used bioinformatics tools. Although processing differs for each experiment type, the whole process shares some common steps, summarized in Figure 1.

Public data is downloaded in SRA format and converted to fastq format using the SRA toolkit (14). Subsequently, the quality of the raw sequencing reads are assessed using fastQC (http://www.bioinformatics.babraham.ac.uk/projects/fastqc/) whilst adapters and any overrepresented sequences are trimmed by using trimgalore (http://www.bioinformatics.babraham.ac.uk/projects/trim_galore/). All initial steps of the pipeline are automated, but the resulting reports are manually inspected and any samples failing at any of these steps may be discarded.

Once the raw reads have been processed, the pipeline continues with the alignment step. To allow samples from the same species to be directly compared, experiments (mouse or human) are always aligned to the same reference genome: GRCm38 (mm10) for mouse and GRCh37 (hg19) for human. Read alignment for ChIP-Seq and DNase-Seq is performed using bowtie2 (17), the reads from the DNase-Seq experiments, are mapped to a 75 bp binned version of the mapping genome, as proposed by John *et al.* (18), whilst ChIP-Seq data is directly mapped to the corresponding genome. Spliced Transcripts Alignment to a Reference (STAR) (19) is used for the alignment of the RNA-Seq reads to the whole genome. Both processing methods generate a Sequence Alignment/Map (SAM) format file of uniquely aligned reads and at this stage, samples with a low number of uniquely mappable reads may be discarded.

Next, using SAMtools (20) the SAM files are transformed into an intermediate Browser Extensible Data (BED) file that allows conversion to a bigWig format (read density profile) (hgdowload.cse.ucsc.edu/admin/exe/bedGraphtoBigWig). During the BED to bigWig transition, ChIP-Seq experiment reads are extended forward to be 200 bp. Depending on the experiment type, additional processing steps are undertaken, for TF ChIP-Seq experiments, peaks (putative binding sites) are called using macs2 (20) for a range of *P*-value stringencies (from 10^−2^ to 10^−15^) by comparing the experimental sample with the appropriate control (where available). At this stage, every stringency is manually inspected and an optimum *P*-value established. The resulting peak profiles are then transformed into a BED format with a uniform length of 400 bp (with the summit of TF binding in the centre ±200 bp).

For RNA-Seq experiments, HTSeq (http://www-huber.embl.de/users/anders/HTSeq/doc/overview.html) is used, considering only exome-aligned reads using the UCSC transcriptome model, which for a given set of replicates, computes a single read count profile. This generates a text file with genes as rows and sample(s) as columns, with each element being the number of reads mapped to each gene (considered as the union of all its exons).

## DATABASE FEATURES AND ANALYSIS TOOLS

**Figure 2.**
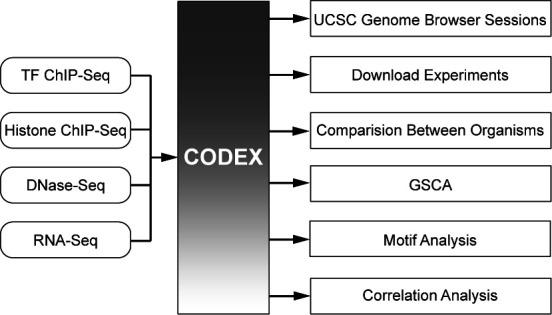
User-friendly, comparative and informative analysis of NGS data. CODEX provides users with immediate access to uniformly analysed publicly available TF ChIP-Seq, histone ChIP-Seq, DNase-Seq and RNA-Seq experiments. NGS experiments can be viewed as sessions in the UCSC Genome Browser, downloaded for further analysis or further integrated using built-in web-tools including Comparison Between Organisms, GSCA, Correlation Analysis or Motif Analysis.

CODEX provides an easy and flexible framework to access NGS data through a user-friendly web interface, and additionally supports several useful web-tools for on-the-fly analysis of NGS experiments (Figure 2).

### Selection and data visualization

The home page of CODEX provides immediate access to NGS experiments of interest through simple filtering, by organism and/or any of the specialized repositories. Users can then browse the experiments and specifically select those of interest. Alternatively, the database can be searched by cell type or TF. Selected experiments are stored into anonymous sessions, allowing the user to re-navigate the site without losing previously selected experiments; up to five different datasets can be managed simultaneously. Although sessions will expire after 1 h of inactivity, users may recover them by generating a sharing link.

For any number of samples contained within CODEX, users are able to visualize density profiles immediately online using the UCSC Genome Browser (21). For TF ChIP-Seq experiments, sample peak profiles or a combined version of density and peak profiles can be visualized. CODEX also provides the line-commands of each single sample track to allow users to set up their own custom UCSC Genome Browser sessions.

### Gene quest

An alternative way to explore TF ChIP-Seq experiments in CODEX is by querying the database for peak-to-gene associations. As mentioned above, peak profiles are determined for all TF ChIP-Seq samples. The Bioconductor ChIPpeakAnno package (22) is used to establish associations between these peaks and putative TF target genes. Gene Quest allows users to search the entire database, to find all the factors associated with a set of genes or, inversely find all the target genes of any factor.

### Correlation and gene set control analysis tools

CODEX includes two built-in web-tools for performing on-the-fly analysis of selected ChIP-Seq experiments: Correlation Analysis and Gene Set Control Analysis (GSCA) (23).

The CODEX Correlation Analysis tool allows users to compare the peak profiles between selected ChIP-Seq experiments. This analysis converts peak profiles into binary vectors within a matrix, with columns as experiments and rows as genomic elements. Where a peak is called at a genomic region, the event is given a value of one, while no peak is annotated as zero. As peak profile matrices are represented by a large number of zero elements, the Pearson's correlation tends to provide a negative close-to-zero coefficient and fails to identify binding profile correlations, even if experiments share a significant number of peak regions. We have therefore opted to use the Dice coefficient to compute similarity, which only takes into account for the calculus the bound regions. The results from this analysis can either be displayed as heat map showing coefficient of agreement or percentage overlap.

GSCA provides a complementary computational approach to the routine Gene Ontology analysis (23). This web-tool uses the peak-to-gene association analysis to identify over-represented overlaps between an inputted set of genes and TF target genes, from selected experiments.

### Motif analysis

CODEX provides pre-computed *de novo* motif discovery analysis using Homer (24) and TOMTOM (25). For each ChIP-Seq experiment in CODEX, peak profiles are used as inputs for Homer to identify consensus sequences that are enriched in the sample relative to background (randomly selected sequences from the genome). CODEX provides an HTML page of the results and motif position weight matrices (PWM) for each TF experiment. CODEX also displays results from an independent search conducted by TOMTOM, using a larger set of known motifs which were manually curated in-house from publications and public resources (Jaspar, UniProbe and Jolma *et al.* (26–28)). PWMs of enriched motifs found by Homer that passed the quality threshold (*P*-value ≤ 10^−10^ and % target ≥ 5%) are converted to Multiple Em for Motif Elicitation (MEME) format and used as input to TOMTOM. Significant similarities of enriched motifs to known motifs are reported in HTML output within CODEX.

### Comparison of NGS datasets between organisms

As experiments from different organisms are not directly comparable within a UCSC Genome Browser session, we have developed a web-tool that identifies common TF target genes for experiments from different organisms. The web-tool computes the intersection of the two TF target gene lists using the NCBI homologene database (29), and provides a text file with the common gene names and EntrezGene identifiers (30).

### Downloading processed data

CODEX provides free access to all processed and pre-computed analysis from the built-in web-tools. All intermediate and final result files are freely available from CODEX, so users can download them and perform any further custom analysis. For all NGS experiments in CODEX, we provide the alignment quality reports (FastQC) and the aligned-reads density profiles (bigWig). For ChIP-Seq experiments, CODEX additionally provides the peak files (BED) and lists of TF target gene (genes). For RNA-Seq, CODEX also supplies the binary mapped-reads files (BAM) and the lists of the number of counts per gene (counts).

### Analysis and integration of unpublished private NGS data

CODEX also provides a framework to store private data The treatment of unpublished data must follow the same guidelines as the public data using the same pipeline, because once published, experiments will become publicly available in CODEX. Users must therefore register to become a CODEX-consortium member and agree to the standardized pipeline described above, before being provided access. When registered users are logged in, their private samples are displayed along with the rest of the public experiments, and may use all the features, analyses and web-tools described above.

## DISCUSSION AND FUTURE IMPROVEMENTS

The standardized bioinformatic data processing, expert manual curation, user-friendly web-interface and built-in web-tools make CODEX a highly relevant resource for both experimental and computational biologists alike. The ability to instantly access the transcriptomes and epigenomes of numerous different cell types and disease states affords a unique opportunity to drive novel biological discoveries and clinical insights. We have endeavoured to produce an interface that is user-friendly and a database that is valuable to the general scientific community. By manually curating the details for every experiment so as to contain only clear and concise information, the cell-type, derivation and treatment of each sample can be quickly understood. With several simple selections, users can immediately visualize multiple experiments in the UCSC Genome Browser or take advantage of the bioinformatic web-tools available. In particular, we envision CODEX helping experimental biologists with otherwise difficult or time-consuming comparisons, for example:

- Comparison of a users ChIP-Seq experiment for a factor which exists within CODEX to all similar experiments within the database and ask how the binding of this TF compares across cell types, treatments or disease states.
- To determine the similarity between an experiment for a factor not contained within CODEX, peak files from experiments of interest can be downloaded and easily compared to the peak binding files of the individuals experiment and the overlap of the experiments calculated.

CODEX, which currently holds over 1000 ChIP-Seq, DNase-Seq and RNA-Seq samples, evolved from HAEMCODE (31), a mouse haematopoietic ChIP-Seq only database with ∼300 datasets in 2013, and which itself evolved from a smaller compendium of 53 ChIP-Seq samples (32) from 2011. The evolution of these databases highlights the rapidity of NGS data generation and efforts to overcome the challenges of collecting, analysing and hosting such data to provide a useful and up-to-date community resource. CODEX also encompasses and goes beyond other specialized or consortium-specific databases such as BloodChIP (34), hmChIP (35) or Factorbook (36). However, as CODEX has to date focused on haematopoietic and ES cell types, samples from other cell types held by these databases may not be shared by CODEX, and the hosted web-tools differ between databases. As other areas of biology move towards large-scale NGS data generation, new specialized repositories can readily be added to CODEX. To further help CODEX stay up-to-date, additional web crawling of ENCODE and BLUEPRINT websites will have the potential to incorporate other publicly available but yet unpublished datasets from these projects.

Development of new web-tools, such as those to further compare DNA motif enrichment and TF binding sites, or interrogate samples from different organisms, will help further our understanding of the conservation of transcriptional regulation at the organ, organism and evolutionary level. Additionally, as more RNA-Seq datasets are published, we aim to further integrate TF ChIP-Seq and RNA-Seq datasets to help uncover combinatorial and cell-type specific TF activities and characterize core transcriptional programmes that define cellular identity. Such datasets and web-tools will make microarray databases, such as BloodExpress (33), all but redundant.

CODEX provides ‘small science’ with the opportunity to mine the wealth of data generated by the wider scientific community and large-scale NGS projects. Interrogation of such large datasets will help drive our understanding of mammalian development, homeostasis and disease, and build towards a unified understanding of these processes.
